# High-Throughput Sequencing Reveals the Differential MicroRNA Expression Profiles of Human Gastric Cancer SGC7901 Cell Xenograft Nude Mouse Models Treated with Traditional Chinese Medicine Si Jun Zi Tang Decoction

**DOI:** 10.1155/2021/6119212

**Published:** 2021-08-17

**Authors:** Junfu Guo, Xiangnan Li, Lanying Miao, Hongwei Sun, Xia Gao, Shengnan Guo, Yueshi Zhang, Peiwei Cong, Wenna Chen

**Affiliations:** ^1^Teaching and Experiment Center, Liaoning University of Traditional Chinese Medicine, Shenyang 110847, Liaoning, China; ^2^Department of Laboratory, Second Affiliated Hospital of Dalian Medical University, Dalian 116027, Liaoning, China; ^3^Department of Medical Laboratory Science, Liaoning University of Traditional Chinese Medicine, Shenyang 110847, Liaoning, China

## Abstract

*Objectiv*e. The present study aimed to investigate the potential mechanism underlying the antitumor effect of Si Jun Zi Tang (SJZT) decoction on gastric cancer. *Methods.* Twelve human gastric cancer SGC7901 cell xenograft nude mouse models were established. The mice were randomly divided into the Model group and SJZT group. SJZT exerted significant antitumor effects after 21 days of decoction administration. High-throughput sequencing was used to analyze the microRNA (miRNA) expression profiles of tumor tissues. Bioinformatics analysis was performed to provide further information regarding the differentially expressed miRNAs. Five representative differentially expressed miRNAs and four predicted target genes were further validated using quantitative real-time reverse transcription PCR (qRT-PCR). *Results.* We identified 33 miRNAs that were differentially expressed in the SJZT group compared with the Model group. Among them, 32 miRNAs were upregulated and 1 miRNA was downregulated. Bioinformatic analysis showed that most of miRNAs acted as tumor suppressors and their target genes participated in multiple signaling pathways, including the PI3K/Akt signaling pathway, microRNAs in cancer, and Wnt signaling pathway. The qRT-PCR result confirmed that miR-223-3p, miR-205-5p, miR-147b-3p, and miR-223-5p were overexpressed and their respective paired target genes *FUT9*, *POU2F1*, *MUC4*, and *RAB14* mRNA were obviously downregulated in the SJZT group compared with those in the Model group. Network analysis revealed that miR-223-3p and miR-205-5p shared two targets *POU2F1* (encoding POU class 2 homeobox 1) and *FUT9* (encoding fucosyltransferase 9), suggesting they have a common role in certain pathways. *Conclusion.* This study provided novel insights into the anticancer mechanism of SJZT against gastric cancer, which might be partly related to the modulation of miRNA expression and their target pathways in tumors.

## 1. Introduction

Gastric cancer is one of the most common malignant tumors of the digestive system and is the fifth most common cancer and the fourth most common cause of cancer death globally [1]. The incidence and mortality rates of gastric cancer in Eastern countries are the highest, particularly in China, Japan, and South Korea [2]. Although the standardization of radical surgery for gastric cancer and the gradual optimization of comprehensive treatment regimens have improved the overall treatment effect of gastric cancer significantly in recent years, most patients with gastric cancer are faced with the problem of tumor recurrence and metastasis, resulting in poor 5-year survival of advanced gastric cancer [3, 4]. Therefore, it is necessary to identify drugs for treatment of gastric cancer and to explore their potential mechanism of inhibiting recurrence and metastasis.

Among the traditional Chinese medicine (TCM) syndromes that differentiate gastric cancer, the type of spleen deficiency occupies the largest proportion and spleen deficiency can appear in the early stage of gastric cancer and runs through all stages of gastric cancer development. The method of replenishing qi and invigorating the spleen is commonly used in the treatment of gastric cancer in TCM. Si Jun Zi Tang (SJZT), a classic Chinese herbal medicine formula, was originally described in “Tai Ping Hui Min He Ji Ju Fang” (Formulas from the Imperial Pharmacy), which was a book of TCM, officially compiled during the Nang Song dynasty about a thousand years ago. SJZT is composed of four different herbs, including *Radix Ginseng* (Renshen), *Poria cocos* (Fuling), *Rhizoma Atractylodis Macrocephala*e (Baizhu), and *Radix Glycyrrhizae* (Gancao) in a ratio of 3 : 3 : 3 : 2. This famous SJZT formula is the basic recipe for replenishing qi and invigorating the spleen and has been used to treat gastric diseases, such as chronic gastritis, gastric ulcers, and gastrointestinal cancer [5–7].

MicroRNAs (miRNAs), 21 to 23 nucleotides in length, are a large class of endogenous noncoding RNAs that exist in many organisms. In humans, they regulate about 30% of gene expression at the posttranscriptional level [8]. Increasing evidence confirms that miRNAs play an important role in diverse tumor-related biological processes, such as cell proliferation, differentiation, apoptosis, invasion, and stemness in gastric cancer [9, 10].

Therefore, in the present study, we performed miRNA sequencing to systematically analyze the differential miRNA expression profiles of human gastric cancer SGC7901 cell xenograft nude mouse models treated with the TCM Si Jun Zi Tang decoction, to further understand the underlying molecule mechanism of the protective effects of SJZT against gastric cancer.

## 2. Materials and Methods

### 2.1. Cells

The human gastric cancer cell line SGC7901 was purchased from the Be Na Culture Collection (BNCC, Shanghai, China) and cultured in Dulbecco's modified Eagle's medium (DMEM; HyClone, Logan, UT, USA) containing 10% fetal bovine serum (FBS; Biological Industries, Beit HaEmek, Israel) supplemented with 1% penicillin-streptomycin (HyClone) at 37°C in a humidified incubator containing 5% CO_2_.

### 2.2. Drugs

The SJZT decoction comprised *Radix Ginseng* (9 g), *Poria coco*s (9 g), *Rhizoma Atractylodis Macrocephala*e (9 g), and *Radix Glycyrrhizae* (6 g). All crude herbs were purchased from the pharmacy of the First Affiliated Hospital, Liaoning University of Traditional Chinese Medicine (Shenyang, China).

### 2.3. Mice and Xenografts

BALB/C nude male mice (*n* = 12, 6−8 weeks old, weighting 20 ± 2 g) were purchased from the Beijing HuaFuKang Biotechnology Co., Ltd. (Beijing, China). Mice were fed under specific pathogen-free conditions that strictly followed these conditions: temperature, 22–26°C; humidity, 40–60%; and a 12 h dark-light cycle. All animals were allowed free access to water and irradiated food for 1 week before the experiments. SGC7901 cells were harvested to prepare cell suspensions containing 1 × 10^7^ cells/ml, which were injected into the right flanks of the nude mice at 200 *µ*l/mouse, respectively. When the mean tumor volume reached approximately 100–200 mm^3^, mice with similar tumor sizes were randomly divided into two groups: Model group (saline only) and SJZT group (13.2 g/kg/day SJZT decoction). Six mice were included in each group. Both groups were treated for 3 weeks. The body weight of the mice and their tumor size were measured and recorded every three days. The tumor volume was calculated as volume (mm^3^) = length × width^2^ × 0.5. After 21 days, the animals were sacrificed using anesthesia and their primary tumors were surgically removed and weighed. The tumor-inhibition rates were calculated using the following formula: (W_Model_–W_SJZT_)/W_Model_ × 100%. W_Model_ and W_SJZT_ are tumor weights of the Model and SJZT groups, respectively. One part of each tumor was fixed in 10% formalin for histological analysis. The remaining tumor tissues were rapidly frozen in liquid nitrogen and stored at −80°C for subsequent analyses.

The above experimental protocol was reviewed and approved by the Animal Care and Welfare Committee of the Liaoning University of Traditional Chinese Medicine (approval number: 21010500042019071).

### 2.4. Histological Analysis

Tumor tissues fixed with 10% formalin were dehydrated, embedded in paraffin, and sectioned. The sections were dewaxed as follows: Xylene I (Sinopharm Chemical Reagent Co. Ltd) for 20 min; Xylene II for 20 min; 100% ethanol I (Sinopharm Chemical Reagent Co. Ltd. Shanghai, China) for 5 min; 100% ethanol II for 5 min; and 75% ethanol for 5 min. After rinsing with tap water, the sections were stained with hematoxylin and eosin (H&E) dye solution according to the manufacturer's protocol (Servicebio, Wuhan, China). The sections were then dehydrated as follows: 100% ethanol I for 5 min; 100% ethanol II for 5 min; 100% ethanol III for 5 min; Xylene I for 5 min; and Xylene II for 5 min before being finally sealed with neutral gum. The sections were imaged under a microscope inspection for further analysis.

### 2.5. MicroRNA Sequencing and Analysis

Three tumor tissue samples in each group were delivered to Shanghai Personal Biotechnology Co., Ltd. (Shanghai, China) for miRNA sequencing and analysis [11]. Small RNA libraries were constructed using the NEBNext Multiplex Small RNA Library Prep Set for Illumina (New England Biolabs, Inc., Ipswich, MA, USA) according to the manufacturer's instructions. In brief, 1 *μ*g of total RNA from each sample was ligated to 3′ and 5′ adapters using Ligation Enzyme Mix. The resulting samples were reverse-transcribed using Superscript II reverse transcriptase, followed by PCR amplification. Then, the resultant small RNA libraries were subjected to quality control (QC), which showed that the average size of the inserts was approximately 140 to 150 bp. The sequencing library was then sequenced on a Hiseq platform (Illumina, San Diego, CA, USA) by Shanghai Personal Biotechnology Co. Ltd. DESeq (version 1.39.0, Anders S and Huber W, 2010, Heidelberg, Germany) was used to analyze the differential expression of miRNAs, and differentially expressed mature miRNAs were screened based on following criteria: |log 2(fold change)| ≥ 1 and *p* < 0.05.

### 2.6. Quantitative Real-Time Reverse Transcriptase-Polymerase Chain Reaction (qRT-PCR) of miRNAs

Total RNA was extracted from each sample using a MiPure Cell/Tissue miRNA kit (Vazyme, Nanjing, China) according to the manufacturer's instructions. A reverse transcription reaction was performed using an miRNA 1st Strand cDNA Synthesis kit (by stem-loop PCR) (Vazyme) for selected miRNAs. Then, the newly synthesized cDNA was used as the template for quantitative real-time PCR (qRt-PCR), which was carried out using the miRNA Universal SYBR^®^ qPCR Master Mix (Vazyme). Reactions were processed and analyzed on a Quantstudio-3-Real-Time-PCR system (ThermoFisher Scientific, Waltham, MA, USA). The PCR conditions were 5 min at 95°C, 40 cycles of 95°C for 10 s, and 60°C for 30 s; followed by a melting curve analysis step. All qRT-PCRs were run in triplicate, and data were analyzed according to the comparative Ct (2^−ΔΔCt^) method [12]. The miRNA-specific primers for reverse transcription and qRT-PCR are listed in Table 1. All experiments were carried out independently three times.

### 2.7. Bioinformatics Analysis

The three databases, namely, miRanda (v3.3a), miRDB (v6.0), and miRWalk (v3.0), were used to predict the target genes of the differentially expressed miRNAs. The predicted target genes were analyzed using DAVID online [13, 14]. Gene ontology (GO) was used to categorize the functions of differentially expressed miRNAs. Kyoto Encyclopedia of Genes and Genomes (KEGG) database analysis was used to identify the main biochemical pathways and signaling pathways involving the target genes of the differentially expressed miRNAs.

### 2.8. RNA Extraction and qRT-PCR of mRNA

Total RNA was extracted using a total RNA isolation kit (FOREGENE, Chengdu, China) according to the manufacturer's instructions. A reverse transcription reaction was performed using a First-Strand cDNA synthesis kit (Bimake, Houston, TX, USA) with 1 *μ*g of RNA in a final volume of 10 *μ*l. The newly synthesized cDNA was then used as the template for quantitative real-time PCR (qRT-PCR), which was carried out using 2 × SYBR Green qPCR Master Mix (Bimake). Reactions were processed and analyzed on a Quantstudio-3-Real-time-PCR system (ThermoFisher, Waltham, MA, USA). The PCR conditions were 5 min at 95°C, followed by 45 cycles of 95°C for 15 s and 60°C for 1 min. All qRT-PCRs were run in triplicate, and data were analyzed according to the comparative Ct (2^−ΔΔCt^) method. The qRT-PCR primers were synthesized by Sangon Biotech (Shanghai, China): *FUT9* (forward, CTCTGTGCTGAAAATGAAAAACTT; reverse, TTGTGAGATGGCATCCTTGG), *POU2F1* (forward, CGCAAAATCTTCTAACGCAAC; reverse, GGCTCTGTGGAAGTGTCTGAAT), *MUC4* (forward, AGTAAAAACTACGAGCAGGCGAA; reverse, TTGTAGGCTTCAATCACACGACC), *RAB14* (forward, AAGGAATCTCACCAATCCAAATAC; reverse, ATCTTCTACATTCTCTCCCGTTTT), and *GAPDH* (forward, ATCATCAGCAATGCCTCC; reverse, CATCACGCCACAGTTTCC). Experiments were carried out independently three times.

### 2.9. Statistical Analysis

Data are presented as the mean ± standard deviation. Statistical Product and Service Solutions (SPSS) 17.0 (IBM Corp., Armonk, NY, USA) was used for all statistical analysis. A *t*-test was used to analyze the intergroup differences. A *p* value less than 0.05 was considered statistically significant.

## 3. Results

### 3.1. Effects of SJZT on SGC7901 Cell Xenograft Nude Mouse Models

**Figure 1:**
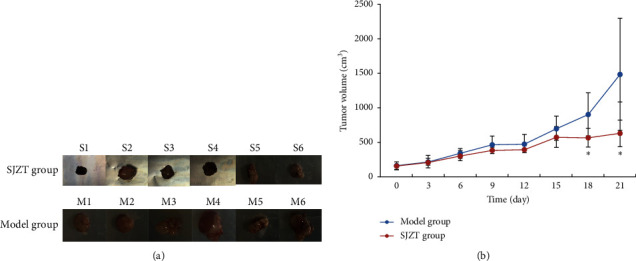
Effect of SJZT on SGC7901 cell xenograft nude mouse models. (a) The digital images of tumors that were treated with SJZT decoction. Mice were given 3 weekly courses of treatment that ended on day 21. (b) The tumor volume growth curve of mice in the model and SJZT groups. *p* < 0.05 vs. the model group.

After tumor formation in nude mice, mice in the Model group and SJZT group were given 3 weekly courses of saline or SJZT decoction, respectively. Three weeks later, the tumor of every mouse exhibited different sizes (Figure 1(a)). SJZT decoction treatment decreased the tumor volume significantly (*p* < 0.05), whereas no significant difference was found in the tumor weight compared with the Model group (*p* = 0.10); the tumor-inhibition rates of SJZT groups were 74.2% (Table 2).The tumor volume curve showed that the tumor volumes of the SJZT group increased significantly more slowly than those of the Model group from the fifteenth day (*p* < 0.05), as shown in Figure 1(b). These results indicated that SJZT has a good antitumor effect.

### 3.2. Histological Analysis of the Tumor Tissue

**Figure 2:**
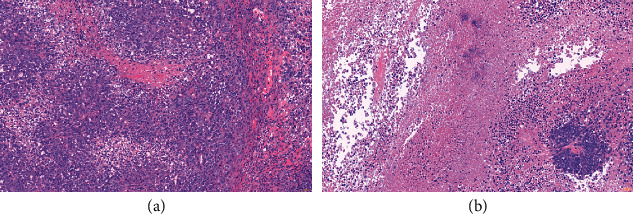
Representative histopathological images of tumor tissues from the (a) model and (b) SJZT groups (200×).

The results of histological analysis displayed that cells in the Model group had marked nuclear pleomorphism, giant nuclei, and visible mitosis (Figure 2(a)). In the SJZT group, the cancer cells exhibited severe necrosis, cancer cell nest shrinkage, and inflammatory cell infiltration (Figure 2(b)).

### 3.3. Effects of SJZT on miRNA Expression Profiles in Tumors

**Figure 3:**
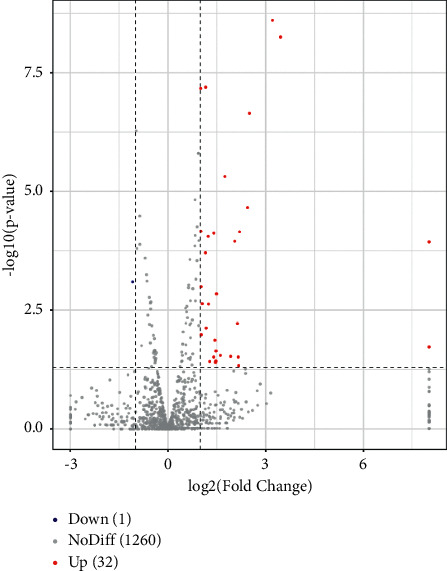
Volcano plot analysis of the differentially expressed miRNAs in the SJZT group relative to the model group. In the differentially expressed miRNA scatter plots, red indicates an upregulated miRNA, green indicates a downregulated miRNA, and grey indicates no significant changes in miRNA expression. The threshold set for significantly differentially expressed miRNAs was |log 2(fold change)| ≥ 1 and *p* < 0.05.

**Figure 4:**
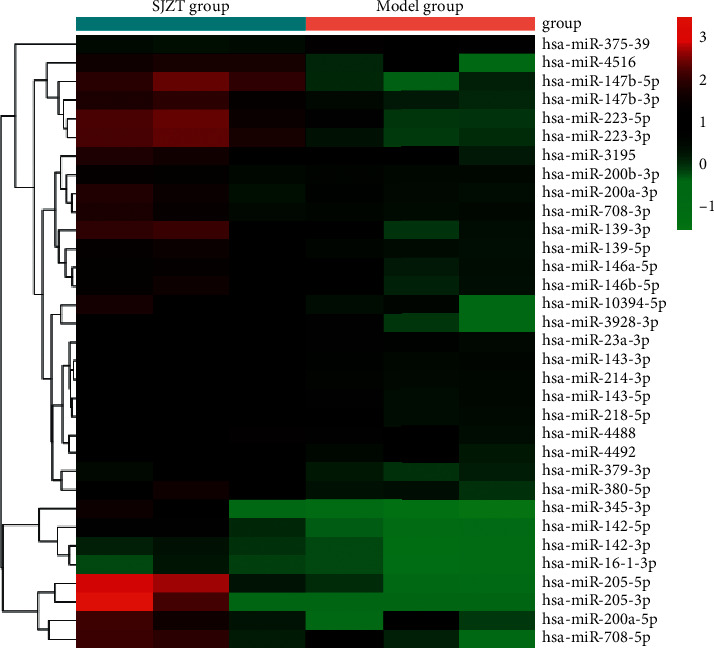
Heatmap and hierarchical clustering of 33 differentially expressed miRNAs in the SJZT group relative to the Model group. Each row represents an miRNA, and each column represents a sample. The color scale shown at the top illustrates the relative expression level of miRNAs; red represents a high relative expression level, and green represents a low relative expression level.

We analyzed the tumor tissue miRNA expression profiles in the different groups. A total of 33 differentially expressed miRNAs (fold change ≥ 2.0, *p* < 0.05) were identified between the SJZT group and the Model group. Among them, 32 were upregulated and 1 was downregulated (Figure 3). The 33 differentially expressed miRNAs are listed in Table 3, and cluster analysis of miRNA expression is shown in Figure 4. These results indicated that SJZT systematically modulated tumor tissue miRNA expression profiles in SGC7901 cell xenograft nude mouse models, and these miRNAs might be important factors that mediate the therapeutic effects of SJZT decoction on gastric cancer.

### 3.4. GO and KEGG Pathway Enrichment Analyses

**Figure 5:**
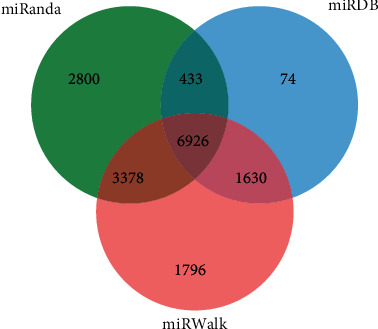
Venn diagram of the target gene counts from miRanda, miRDB, and miRWalk databases. Taking the intersection of the three databases finally resulted in 6926 target genes for further study.

**Figure 6:**
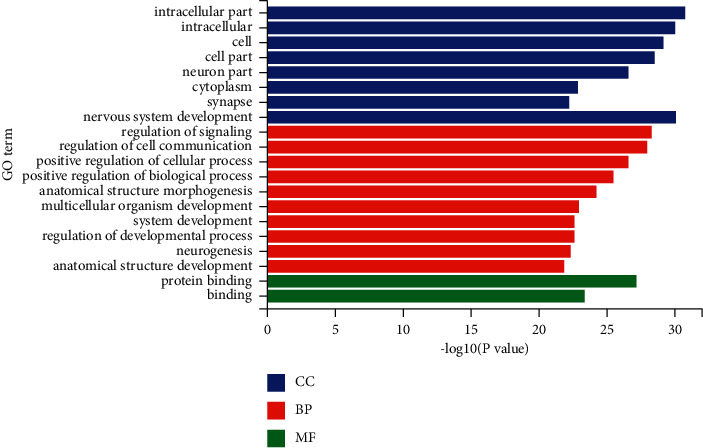
GO analysis for the predicted targets of the differentially expressed miRNAs in the SJZT group relative to the model group (top 20 enriched terms covering biological processes, cellular components, and molecular functions are presented).

**Figure 7:**
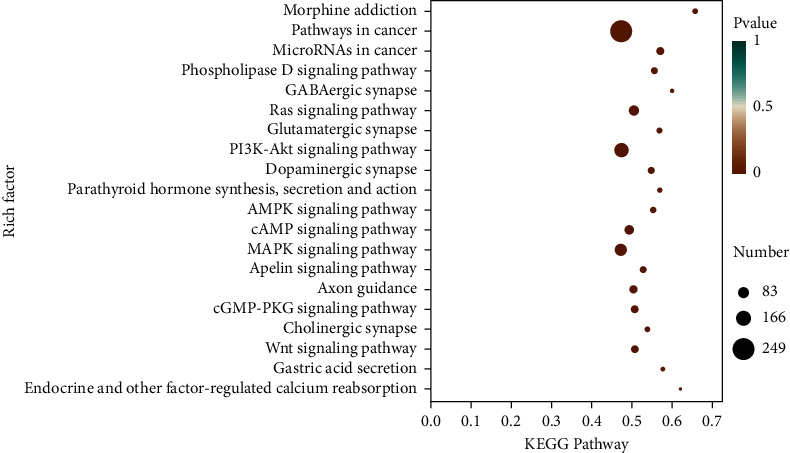
KEGG pathway enrichment analysis of the target genes of the differentially expressed miRNAs in the SJZT group relative to the model group (top 20 enriched pathways). The *x*-axis indicates the proportion of the enriched differential gene in the background genes of the pathway; the *y*-axis indicates the pathway name; point sizes indicate the number of differentially enriched genes; dot colors indicate the size of the *p* value.

The miRanda, miRDB, and miRWalk databases were used to predict the target genes of the 33 differentially expressed miRNAs, and a total of 6,926 target genes from all there databases were identified (Figure 5). To predict the function of the differentially expressed miRNAs, Gene Ontology (GO) functional annotation was used to provide annotations for the candidate target genes in terms of cellular components, biological processes, and molecular functions. We found that “intracellular part,” “nervous system development,” and “protein binding” were the most significantly enriched in the three categories (Figure 6). To analyze the signal transduction and disease pathways that are controlled by the targets of the differentially expressed miRNAs, the candidate target genes were analyzed using the KEGG pathway. Hypergeometric distribution was used for calculating the target gene enrichment pathway analysis (Figure 7), which showed that the main signaling pathways involving the target genes of the differentially expressed miRNAs included pathway in cancer, microRNAs in cancer, Ras signaling pathway, PI3K-Akt signaling pathway, cAMP signaling pathway, MAPK signaling pathway, Apelin signaling pathway, cGMP-PKG signaling pathway, and Wnt signaling pathway.

### 3.5. Validation of miRNA Expression by qRT-PCR

**Figure 8:**
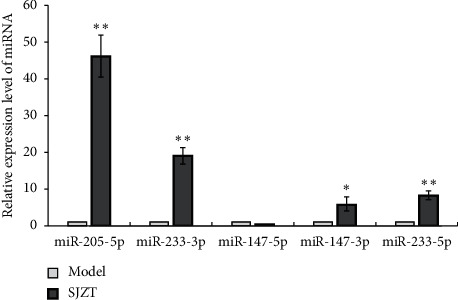
The relative expression level of five selected miRNAs (fold change ≥ 5, *p* < 0.01) was verified using qRT-PCR. Data represent the mean ± SD from three experiments (^*∗*^*p* < 0.05 and ^*∗∗*^*p* < 0.01 vs. the model group).

To validate the findings of miRNA sequencing, we tested the expression levels of five miRNAs (fold change ≥ 5, *p* < 0.01) using qRT-PCR. As shown in Figure 8, miR-147b-5p was significantly downregulated (*p* < 0.05) that was inconsistent with miRNA sequencing data. However, miR-205-5p, miR-223-3p, miR-147b-3p, and miR-223-5p were obviously upregulated after SJZT decoction treatment for 3 weeks (*p* < 0.05 or *p* < 0.01); those were all consistent with the miRNA sequencing data, thus indicating that the sequencing results are reliable.

### 3.6. Regulatory Network Construction and Analyses

**Figure 9:**
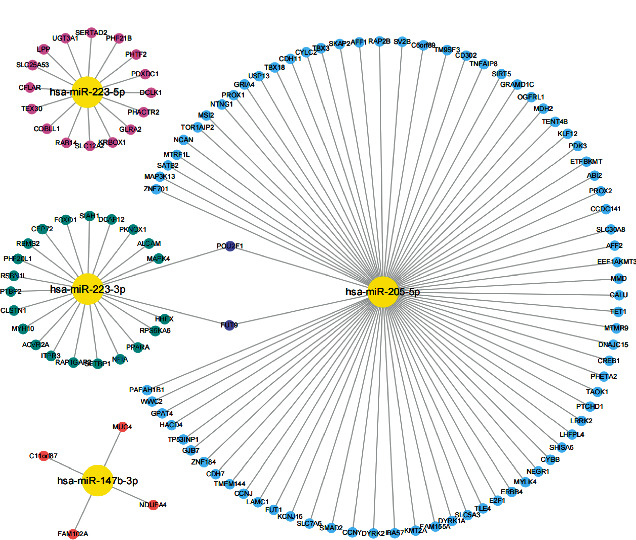
A network of four differentially expressed miRNA (miR-223-3p, miR-147b-3p, miR-223-5p, and miR-205-5p) and their target genes.

Moreover, miRanda, miRDB, and miRWalk databases were used to predict the target genes of the four differentially expressed miRNAs verified by the above qRT-PCR assay and consistent with sequencing data, and a total of 122 target genes were identified. The network of miRNAs and target genes was visualized. It revealed that miR-223-3p and miR-205-5p shared two targets *POU2F1* (encoding POU class 2 homeobox 1) and *FUT9* (encoding fucosyltransferase 9), suggesting they have a common role in some pathways (Figure 9).

### 3.7. Validation of Expression Level of mRNA of Target Genes by qRT-PCR

**Figure 10:**
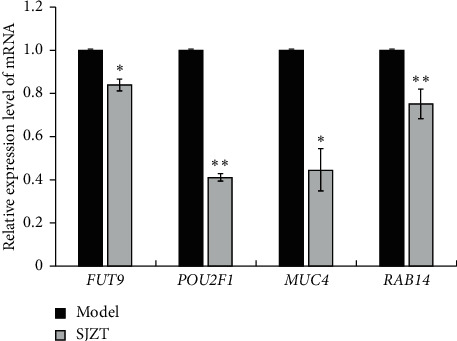
The relative expression level of mRNA of four target genes were verified using qRT-PCR. Data represent the mean ± SD from three experiments (^*∗*^*p* < 0.05 and ^*∗∗*^*p* < 0.01 vs. the model group).

To further validate the predicted result, we tested the expression levels of mRNA of four target genes by qRT-PCR. As shown in Figure 10, *FUT9*, *POU2F1*, *MUC4*, and *RAB14* mRNA were obviously downregulated after SJZT decoction treatment (*p* < 0.05 or *p* < 0.01), suggesting SJZT plays an antitumor role by upregulating the expression of miRNAs to inhibit the expression of their target genes.

## 4. Discussion

In recent years, a large number of studies have shown that TCM plays an important role in prolonging the survival time of patients with malignant tumors, improving their quality of life and reducing the risks of tumor recurrence and metastasis [15, 16]. TCM theories propose that the functions of the spleen and stomach are closely related to the occurrence of various diseases [17]. SJZT is one of the most famous invigorating spleen therapy recipes in TCM. Research showed that modified SJZT could inhibit colorectal cancer liver metastasis by activating the innate immune system [18]. An alcohol extract of SJZT exerted antimelanoma effects and regulated the miR-34b/c-Met/*β*-catenin pathway in a melanoma mouse model [19]. Li et al. [20] found that SJZT could enhance the anticancer effects of gefitinib in lung cancer using metabolomics and network pharmacology analyses. In the present study, gastric gavage administration of SJZT inhibited the xenograft tumor growth of gastric cancer significantly in nude mice, demonstrating *in vivo* evidence of antitumor activity. During the whole experimental period, the tumors grew rapidly in the Model group and, eventually, the average tumor volume reached 902.89 mm^3^, while in the SJZT group, the tumor volume only reached 410.63 mm^3^. The H&E staining illustrated that SJZT had an excellent antitumor activity.

The role of miRNAs as key regulators that control a wide variety of fundamental cellular processes is increasingly recognized in almost all biology and biomedicine fields [21]. The distinct characteristics of TCM formula may be explained, in part, by the induction of differential miRNA expression. Chen et al. [22] confirmed that Yangzheng Sanjie decoction containing serum could inhibit the proliferation and induce the apoptosis of gastric cancer cells mediated by miR-7-targeting *EGFR* (encoding the epidermal growth factor receptor). Pan et al. [23] investigated the potential mechanism underlying the protective effect of Shenling Baizhu San on nonalcoholic fatty liver disease by microRNA sequencing and identified 28 differentially expressed miRNAs. To enrich the evidence for SJZT's antitumor effect at the level of posttranscriptional gene regulation, we performed miRNA sequencing and found 33 miRNAs that displayed differential expression patterns in the SJZT group compared with Model group, demonstrating a potent effect of SJZT in globally altering tumor miRNA expression.

To further study the differentially expressed miRNAs' biology functions, we predicted their target genes, which identified 6,926 target genes predicted by three databases. GO and KEGG pathway analyses revealed that these genes were enriched in many important signaling pathways, especially several key cancer-related signaling pathways including the PI3K/Akt signaling pathway, microRNAs in cancer, and Wnt signaling pathway, which have been reported to be closely associated with tumor initiation.

Indeed, previous studies have shown that gastric cancer patients with simultaneous expression of PI3K/p-AKT/p-mTOR had worse outcome [24]. Furthermore, calcium release-activated calcium modulator 2 mediated store-operated calcium activity and promoted gastric cancer tumorigenic properties through the activation of the PI3K/Akt signaling pathways [25]. miR-489 functions as an oncogene can significantly promote GC cell (SGC7901 and MKN45) proliferation, invasion, and migration and effectively block the activation of PI3K/AKT pathway [26]. Yu et al. found that Zi Yin Hua Tan (ZYHT) recipe could inhibit tumor growth and cell proliferation and promote apoptosis in gastric cancer by suppressing the PI3K/AKT pathway [27]. Liu et al. confirmed that ginsenoside Rg3 (Rg3), an herbal medicine, also a component of SJZT, can ameliorate gastric precancerous lesions in Atp4a-/- mice via inhibition of glycolysis through the PI3K/AKT pathway [28]. Recent research showed that ginsenoside might possibly deliver their beneficial effects through the modulation of miRNA expression in various tumors [29–31]. It is suggested that ginsenoside, which is a component of SJZT, may inactivate PI3K/AKT signaling pathway by regulating miRNA expression to exert antitumor effect.

The downregulation of specific miRNAs could result in an increase in oncogene expression and induce subsequent malignant effects on cell proliferation, differentiation, and apoptosis that lead to tumor growth and progress. Those miRNAs act as tumor suppressors. As expected, many differentially expressed miRNAs (upregulated after SJZT treatment in the present study) function as tumor suppressors and are downregulated in gastric cancer tissues, such as miR-143-3p [32], miR-139-5p [33], miR-142-5p [34], miR-218-5p [35], and miRNA-146a-5p [36].

The Wnt signaling pathway regulates both embryonic development and maintains adult tissue homeostasis [37]. Wnt components are dysregulated in many tumors and control several fundamental cell functions, including proliferation, invasion, migration, and stemness. SERPINH1 can promote survival, invasion, and migration of human gastric cancer SGC7901 cells via the Wnt/*β*-catenin signaling pathway [38]. Jung et al. confirmed that HER2-overexpressing gastric cancer cells exhibited increased stemness and invasiveness and were regulated by Wnt/*β*-catenin signaling [39]. miR-338 can inhibit EMT of gastric cancer cells through deactivation of Wnt/*β*-catenin signaling, targeting at EphA2 [40]. Jiang et al. illustrated that Maimendong and Qianjinweijing Tang (Jin formula) could suppress the development of lung cancer by upregulating miR-149-3p to inhibit Wnt/*β*-catenin axis [37].

In addition, four differentially expressed miRNAs (miR-223-3p, miR-205-5p, miR-147b-3p, and miR-223-5p) from the sequencing results and their respective paired target genes (*FUT9*, *POU2F1*, *MUC4*, and *RAB14*) were validated by qRT-PCR assays. The qRT-PCR results were consistent with the miRNA sequencing data and the predicted result. Previous studies have shown that miR-223-3p functioned as a tumor suppressor in several different cancers, including glioblastomas, breast cancer, and oral squamous cell carcinoma [41–44]. Dou et al. [45] confirmed that miR-223-5p suppressed tumor growth and metastasis in non-small-cell lung cancer by targeting *E2F8* (encoding E2F transcription factor 8). Recent research also showed that miR-223-5p targets *ERG* (encoding ETS transcription factor ERG) and inhibited prostate cancer cell proliferation and migration [46]. In addition, miR-205-5p expression in breast cancer tissues and cell lines was decreased compared with that in normal tissues and a normal cell line. Overexpression of miR-205-5p significantly augmented the cytotoxic effects of gemcitabine treatment in MDA-MB-231 and BT549 cells [47].

To further analyze the functions of the differentially expressed miRNAs, we used three databases to predict the target genes of the above four selected miRNAs that were validated by qRT-PCR and construct miRNA target gene networks. The network of the 4 miRNAs and their 122 target genes was visualized. The results showed that the four miRNAs regulated the expression of multiple genes, among which miRNA-205-5p had dozens of potential gene targets, whereas miRNA-147b-3p had the fewest targets, which might bring new direction and new ideas for subsequent studies. Notably, miR-223-3p and miR-205-5p share 2 targets POU2F1 and FUT9. POU2F1, also named “octamer-binding transcription factor-1” (OCT1), is related to OCT4, an embryonic stem cell master transcription factor. OCT1 is overexpressed in some forms of gastric cancer [48, 49]. OCT1 can regulate normal and cancer stem cell functions [50]. OCT1 loss can reduce oncogenic transformation *in vitro* and tumorigenicity *in vivo* [51]. Blanas et al. confirmed that FUT9 overexpression could increase Lewis x, SOX2, ALDH, and CD44 expression, tumor sphere formation, resistance to 5-FU treatment, and *in vivo* tumor growth [52]. Auslander et al. found that *FUT9* silencing could decrease the expression level of tumor-initiating cell (TIC) markers CD44 and OCT4 in colon cancer cells [53]. Research has shown that miR-205-5p overexpression could inhibit the drug resistance, proliferation, and colony formation rate, while promoting the apoptosis of gallbladder cancer stem cells [54]. Chaudhary et al. confirmed that lentivirus-mediated overexpression of miR-205 could inhibit the proliferation of pancreatic cancer stem cells and tumor growth in mouse models [55]. These considerations further suggested the effect of SJZT on gastric cancer stem cells. Furthermore, MUC4, encoding a large transmembrane mucin, is often overexpressed in various epithelial malignancies, such as pancreatic cancer, prostate cancer, lung cancer, and breast cancer, and is thought to play an important role in tumor cell biology [56]. Recent studies illustrated that MUC4 was correlated with prognosis of gastric cancer patients [57] and promoted proliferation and invasion of gastric cancer cells [58]. Guo et al. confirmed that RAB14 functioned as a novel tumor oncogene in gastric cancer. Overexpressed RAB14 can induce SGC7901 cell proliferation and promote cell apoptosis [59]. Li et al. [60] found that miR-320a influenced proliferation and cell cycle of gastric cancer cells by targeting RAB14. Therefore, except stemness, SJZT may also affect other aspects of gastric cancer by regulating miRNA and paired target genes, which is worth additional investigation in the future studies.

## 5. Conclusion

In the present study, we first established a human gastric cancer SGC7901 cell xenograft nude mouse model. After SJZT administration, an obvious antitumor effect was observed.

Subsequently, high-throughput sequencing was used to reveal the differential microRNA expression profiles of tumor tissues. Bioinformatics analysis was performed to provide further information on the mechanism of the effects of SJZT on gastric cancer. The results showed that SJZT decoction altered miRNA expression profiles to inhibit the expression of various genes, thus affecting biological functions of gastric cancer cells including proliferation, apoptosis, migration, and cancer stem cell characteristics, thereby influencing the progression of gastric cancer. This study provided novel insights into the anticancer mechanism of SJZT against gastric cancer, which might be partly related to the modulation of tumor miRNA expression and its target pathways. However, considering the limited sample number in this study, the miRNA expression profiles may not be entirely accurate. Every differentially expressed miRNA should be validated by qRT-PCR assays. Furthermore, related *in vitro* experiments will be carried out to support the bioinformatics analysis.

## Figures and Tables

**Table 1:** MiRNA primer sequences.

miRNA	Reverse transcription primer sequence (5′-3′)	qRT-PCR primer sequence (5′-3′)
hsa-miR-205-5p	GTCGTATCCAGTGCAGGGTCCGAGGTATTCGCACTGGATACGACCAGACT	CGTCCTTCATTCCACCGG

hsa-miR-223-3p	GTCGTATCCAGTGCAGGGTCCGAGGTATTCGCACTGGATACGACTGGGGT	GCGCGTGTCAGTTTGTCAAAT

hsa-miR-147b-5p	GTCGTATCCAGTGCAGGGTCCGAGGTATTCGCACTGGATACGACAGTTTG	CGCGTGGAAACATTTCTGCA

hsa-miR-147b-3p	GTCGTATCCAGTGCAGGGTCCGAGGTATTCGCACTGGATACGACAGCAGA	CGGTGTGCGGAAATGCT

hsa-miR-223-5p	GTCGTATCCAGTGCAGGGTCCGAGGTATTCGCACTGGATACGACAACTCA	CGCGCGTGTATTTGACAAGC

**Table 2:** Effects of SJZT on the tumor volume and weight of SGC7901 cell xenograft nude mice models.

Group	*n*	Volume (cm^3^)	Weight (g)	Tumor-inhibition rates (%)
Model	7	902.89 ± 491.24	0.96 ± 0.86	—
SJZT	7	410.63 ± 210.43^*∗*^	0.25 ± 0.16	74.13

*Note*. ^*∗*^*p* < 0.05 vs. the Model group.

**Table 3:** Differentially expressed miRNAs in the SJZT group relative to the Model group.

No.	miRNA	Expression type	Fold change	*p* value
1	hsa-miR-205-3p	Up	Infinity	0.018706
2	hsa-miR-205-5p	Up	259.6664	0.000115
3	hsa-miR-223-3p	Up	10.98984	5.60*E* − 09
4	hsa-miR-147b-5p	Up	9.275823	2.48*E* − 09
5	hsa-miR-147b-3p	Up	5.675385	2.26*E* − 07
6	hsa-miR-223-5p	Up	5.433369	2.18*E* − 05
7	hsa-miR-142-5p	Up	4.59781	7.02*E* − 05
8	hsa-miR-200a-3p	Up	4.504338	0.046485
9	hsa-miR-708-5p	Up	4.459526	0.03048
10	hsa-miR-345-3p	Up	4.378069	0.00603
11	hsa-miR-4516	Up	4.140227	0.000111
12	hsa-miR-200a-5p	Up	3.794401	0.029563
13	hsa-miR-139-5p	Up	3.346087	4.82*E* − 06
14	hsa-miR-200b-3p	Up	3.046158	0.028182
15	hsa-miR-146a-5p	Up	2.815306	0.001432
16	hsa-miR-139-3p	Up	2.786198	0.022766
17	hsa-miR-708-3p	Up	2.776734	0.037235
18	hsa-miR-3928-3p	Up	2.756557	0.039446
19	hsa-miR-380-5p	Up	2.71972	0.013451
20	hsa-miR-142-3p	Up	2.659047	7.46*E* − 05
21	hsa-miR-10394-5p	Up	2.652237	0.03049
22	hsa-miR-379-3p	Up	2.436079	0.037967
23	hsa-miR-146b-5p	Up	2.370031	0.002351
24	hsa-miR-218-5p	Up	2.353085	8.71*E* − 05
25	hsa-miR-3195	Up	2.256784	0.007502
26	hsa-miR-214-3p	Up	2.234376	6.35*E* − 08
27	hsa-miR-4488	Up	2.224693	0.000194
28	hsa-miR-4492	Up	2.077164	0.002314
29	hsa-miR-16-1-3p	Up	2.032917	0.010334
30	hsa-miR-143-5p	Up	2.026165	0.001016
31	hsa-miR-23a-3p	Up	2.019862	6.82*E* − 05
32	hsa-miR-143-3p	Up	2.012136	6.72*E* − 08
33	hsa-miR-375-3p	Down	0.472281	0.000799

## Data Availability

The datasets used and analyzed during the current study are available from the corresponding author upon reasonable request.
